# Comparison of coercive practices in worldwide mental healthcare: overcoming difficulties resulting from variations in monitoring strategies

**DOI:** 10.1192/bjo.2023.613

**Published:** 2024-01-11

**Authors:** Martha K. Savage, Peter Lepping, Giles Newton-Howes, Richard Arnold, Vincent S. Staggs, Steven Kisely, Toshio Hasegawa, Keith S. Reid, Eric O. Noorthoorn

**Affiliations:** School of Geography, Environment and Earth Sciences, Victoria University of Wellington, Wellington, New Zealand; Centre for Mental Health and Society, Wrexham Academic Unit, Bangor University, Bangor, UK; University of Otago, Wellington, New Zealand; School of Mathematics and Statistics, Victoria University of Wellington, Wellington, New Zealand; University of Missouri-Kansas City and Children's Mercy Research Institute, Kansas City, Missouri, USA (now at IDDI Inc, Raleigh, North Carolina, USA); The University of Queensland, Brisbane, Australia; Department of Occupational Therapy, Faculty of Health Sciences, Kyorin University, Mitaka, Japan; Health and Life Sciences, Northumbria University, Newcastle upon Tyne, UK; and Cumbria, Northumberland, Tyne and Wear NHS Foundation Trust, Newcastle upon Tyne, UK; Radboud University Nijmegen, Nijmegen, the Netherlands; and Ggnet Mental Health Trust Warnsveld, Warnsveld, The Netherlands

**Keywords:** Epidemiology, human rights, consent and capacity, ethics, in-patient treatment

## Abstract

**Background:**

Coercive or restrictive practices such as compulsory admission, involuntary medication, seclusion and restraint impinge on individual autonomy. International consensus mandates reduction or elimination of restrictive practices in mental healthcare. To achieve this requires knowledge of the extent of these practices.

**Aims:**

We determined rates of coercive practices and compared them across countries.

**Method:**

We identified nine country- or region-wide data-sets of rates and durations of restrictive practices in Australia, England, Germany, Ireland, Japan, New Zealand, The Netherlands, the USA and Wales. We compared the data-sets with each other and with mental healthcare indicators in World Health Organization and Organisation for Economic Cooperation and Development reports.

**Results:**

The types and definitions of reported coercive practices varied considerably. Reported rates were highly variable, poorly reported and tracked using a diverse array of measures. However, we were able to combine duration measures to examine numbers of restrictive practices per year per 100 000 population for each country. The rates and durations of seclusion and restraint differed by factors of more than 100 between countries, with Japan showing a particularly high number of restraints.

**Conclusions:**

We recommend a common set of international measures, so that finer comparisons within and between countries can be made, and monitoring of trends to see whether alternatives to restraint are successful. These measurements should include information about the total numbers, durations and rates of coercive measures. We urge the World Health Organization to include these measures in their Mental Health Atlas.

There is considerable debate on how much the United Nations Convention on the Rights of Persons with Disabilities should apply to psychiatric care, particularly about the issue of coercion.^1^ Most psychiatrists agree that coercive practices should be used rarely, as a last resort, and for as short a time as possible. To address this concern, the World Psychiatric Association (WPA) and the World Health Organization (WHO) have worked together on alternatives to coercion.^2,3^ Here, we consider coercive practices to include the restrictive practice incidents (RPI) of seclusion and physical (also known as manual), mechanical and chemical restraint; and involuntary admission, because it occurs without the consent of the person being admitted.^4^ The primary intent of this study was to investigate the relative use of coercive practices across various countries. To do so, standardised measures are necessary.

## The importance of monitoring

The effectiveness of efforts to replace coercion with better alternatives can only be evaluated by monitoring how often coercive practices are used and whether use decreases over time. As restrictive practices are often considered to be a failure of care, they are reportable as an ‘incident’, as a fall or occupational injury would be.^5^ Similar to accident investigations, a debriefing after each incident with the relevant people is one way to minimise future occurrences.^6^ However, there are limited data on the use of coercive measures, with most published studies restricted to a small number of hospitals^7^ or countries.^8^ Moreover, existing coercion-tracking measures often vary from country to country or even from hospital to hospital.^8^ It is therefore difficult to determine whether and by how much practices have changed at an international level. Standardisation would allow for more informative comparisons between countries, identification of opportunities for intervention by benchmarking and assessments of any resulting changes in outcomes. The WHO Mental Health Atlas is the only global compilation of data on psychiatric practice.^9^ Only one of its measures, the percentage of people who are involuntarily admitted to hospital, relates to coercion, although it includes indicators of type and quality of care that could correlate with the use of coercion.^9^

Perhaps the first study to compare countries’ use of restraint was that of Steinert et al.^8^ The main recommendation was for the collection of standardised data to allow robust comparisons across countries. More than 10 years later, such standardisation remains rare. Two recent studies developed consistent measures to compare coercive practices across small groups of countries (four in each study).^10,11^ In one, mechanical restraints were calculated in terms of numbers of events per 1 million people per day,^10^ because one of the countries only had information about daily rates. The other considered the use of any restrictive practice in terms of number of events and numbers of people subject to those events per 100 000 population per year,^11^ because different types of restrictive practices seemed to be more common in one country than another. Both found large variations between hospitals within countries. Newton-Howes et al^10^ also found large variations in the incidence of mechanical restraints among countries around the Pacific. For instance, total mechanical restraint use nearly doubled in Japan from 2004 to 2017, although rates decreased among elderly patients after 2017. Rates among the elderly also decreased in Australia and the USA. By contrast, mechanical restraint use increased in New Zealand after 2008, although it remained well below those of the other countries. Lepping et al^11^ found little variation among four European countries in total restraint incidence, but there were large variations in the type of coercive measures used. The present work expands on these studies to show that although finely detailed comparisons across countries may be difficult, approximate comparisons are possible through appropriate conversions of different measures of rates.

## Approach

Although we had initially hoped to assemble all data worldwide to document comparative rates of coercive interventions, the limited availability of data meant we had to restrict the study to countries with readily available data-sets covering most of the country or published multi-country comparisons of mental healthcare. We revised and expanded the comparisons used in our two previous studies^10,11^ and compared involuntary hospital admission, seclusion and restraint use in nine countries. Although countries reported different measures of restraints, such as total numbers of restraints, numbers of people undergoing restraint or hours of restraint use, we standardised the results by normalising by the population of each country and by using information from other sources, such as restraint durations, to convert hours of restraint use to numbers of restraints. We also examined the results in relation to quality of care indicators such as suicide rates, government spending on mental health, and the numbers of different types of mental health professionals per 100 000 population described in the WHO Mental Health Atlas^9^ and the Organisation for Economic Cooperation and Development (OECD) benchmark report^12^ and restraint use detailed in the OECD report.

## Data and method

We compiled readily available data sources for involuntary hospital admission, seclusion, mechanical and physical restraint, and involuntary medication from Australia, England, Germany, Ireland, Japan, The Netherlands, New Zealand, the USA and Wales. All data were anonymised and openly published, and no specific ethics approval was required.

### Population and countries chosen

This analysis reports on population-level data from the nine countries named above. All are high-income countries in the OECD. We chose them because they had readily available regional or national restraint data-sets that included most hospitals that were expected to use restraints.^10,11^ Essentially, this allowed us to capture reported information for the whole of the population in the regions and countries surveyed. Most of these countries have accessible healthcare provided by the state. The databases are described more fully in the Supplementary Text available at https://doi.org/10.1192/bjo.2023.613 and in Table 1.

**Table 1:** Summary of reported measures and calculations to provide uniform rates

Country and reference to main data-set	Raw measurement units and methods tracked (numbers are per year unless otherwise specified)	Information or assumptions in addition to population and raw measurements used to transform to events or admissions per 100 000 population per year (P), events per 1000 bed-days (B), events per 1 000 000 population per day (D) or hours of RPI per 1000 h of patient care (H)
Australia^13^	Events per 1000 bed-days and absolute numbers of events for admissions (separations), involuntary admissions, seclusions, and physical and mechanical restraints. Seclusion durations. States have different reporting requirements and definitions of seclusion and restraints; they are all summed.	All: assume total RPI from sum of types of RPI Admission duration from bed-days/admissions B: nothing extra D: assume daily rate from yearly rate/365 H: as durations of physical and mechanical restraints are unknown, hours of RPI only include seclusion
England^14^	Numbers of people affected by and episodes of admissions, seclusion, mechanical, physical and chemical restraint. Bed-days	All: report was concerned about double-counting. We used RPI from totals from counts of individual types, as they were smaller than the totals reported. Numbers of involuntary admissions from percentages in WHO Atlas.^9^ D: assume daily rate from yearly rate/365 H: no durations of restraint known, so could not calculate
Germany (south-west)^11^	Numbers of people affected by and episodes of admissions, seclusions, mechanical, physical restraints, involuntary medication. Durations of all. Bed-days	P, B: nothing extra D: assume daily rate from yearly rate/365 H: hours of RPI from sum of numbers × durations of each type of RPI. Hours of patient care from bed-days
Ireland^15^	Numbers of people affected by and episodes of seclusion, mechanical and physical restraints, total duration of seclusions, physical restraints and mechanical restraints. Number of beds	P: number of admissions and involuntary admissions from ref.^16^ Duration of admissions from ref.^16^ B: number of bed-days from ref.^16^ D: assume daily rate from yearly rate/365 H: durations of each type of RPI × numbers of each, hours of patient care from bed-days
Japan^17^	Numbers of people on a given day who are admitted voluntarily or involuntarily, and who are mechanically restrained or secluded. Distributions of length of admissions prior to a given day. Another report gives distributions, means and medians of restraint and seclusions for 2 years,^18^ which we averaged.	P: mean durations used to calculate numbers of events and admissions per year B: as in ref.^10^, assumed average bed-days = average number of patients on one day × 365 D: nothing extra H: hours of RPI from numbers of people in RPI on 1 day × 365 × 24, hours of patient care from bed-days
The Netherlands^11,19^	Numbers of events and people affected by seclusion, mechanical restraints, involuntary medication and total RPI. Total admissions, occupied bed-days, total hours of seclusion and mechanical restraints, percentage of people admitted who are secluded	P, B: numbers of involuntary admissions from separate report^20^ D: assume daily rate from yearly rate/365 H: durations of each type of RPI × numbers of each, hours of patient care from bed-days
New Zealand^10,21^	Numbers and mean durations of mechanical restraints and seclusions. Number of people secluded, number of bed-days. Number of patients present in year. Patients who started evaluation for involuntary admission process in the year	P: involuntary admissions assumed to have same percentage of readmissions in year as all admissions. Admissions assumed equal to the number of Health of Nation Outcome Scale (HoNOS) tests administered for the purpose of admission. Number of RPI from sum of mechanical restraints and seclusion B: nothing extra D: assume daily rate from yearly rate/365 H: durations of each type of RPI × numbers of each, hours of patient care from bed-days
United States^22^	Total hours of seclusion and restraint and bed-days in 1 year, and hours of seclusion or restraint per 1000 h of patient care	P, B, D: proportions and durations of types of restraint and seclusion calculated from data-set of restrictive events after violence^23^ and used with hours of patient care to determine numbers of events. Added seclusion and restraint to get RPI H: nothing extra
Wales^11^	Numbers of admissions, seclusion events, number of people secluded and under RPI and percentage of RPI that are seclusion. RPI is only seclusion plus physical restraint. Mean durations of seclusion events and RPI events. Total occupied bed-days	P: percentage of involuntary admissions from ref.^24^ B: nothing extra D: assume daily rate from yearly rate/365 H: durations of each type of RPI × numbers of each, hours of patient care from bed-days

We collected data for adults admitted to acute hospital wards, whether privately or publicly funded. We limited data to acute hospitals because coercion involved in subacute or community-based treatment is of a different nature to that used in hospitals. We attempted to exclude forensic, learning disability and dementia care wards, but this was not possible for all countries. The natures of those services and how they are commissioned and interrelated with other state functions vary among jurisdictions. To determine rates per 100 000 population, we used the entire population of the country or region considered despite our concentration on adult wards; this was because the ages considered in the wards were often not well communicated, and also to ensure that we treated every country or region similarly. The final data-sets used and the populations they included are delineated in the Supplementary Text and Supplementary Spreadsheet.

We began with the WHO Atlas^9^ and with records published by the two studies that directly compared rates of seclusion or restraint in several countries. We used results and data-sets included by Lepping et al,^11^ who examined national or region-wide data-sets of seclusion and physical and mechanical restraint in four European countries (Wales, Ireland, a part of south-west Germany that participated in organised data collection and The Netherlands) for years close to 2013. We also used national mental healthcare data-sets from the respective regions that were examined by Newton-Howes et al^10^ for mechanical restraint in four countries that have borders in the Pacific (Australia, New Zealand, Japan and the USA). The countries all also had data on seclusion, and Australia separately reported mechanical and physical restraints. The original study included years as close as possible to 2017. We used data-sets or documents from each country to include involuntary and voluntary admissions, to expand the data of Newton-Howes et al^10^ to include seclusion and physical restraint, and to separate the restraint types that were combined by Lepping et al.^11^

The WHO Mental Health Atlas^9^ provides reports by individual country as well as summary comparisons broken into geographic regions and income categories. The only coercive measure reported in the Atlas is the number of involuntary hospital admissions. The OECD report^12^ provides some similar measures to those used by the WHO Atlas, as well as reports of restraints and seclusion for OECD countries. The WHO Atlas has the following disclaimer: ‘it is vital to acknowledge the limitations associated with self-reported data, particularly relating to qualitative assessments or judgements (which were often made by a single focal point)’. Therefore, we included involuntary hospital admission figures from country data-sets except in the case of England, for which data were only available in the Atlas.

The WHO data are reported only for the whole of Great Britain (England, Scotland and Wales). We added England to our previous analysis of Wales^11^ to include more of Great Britain. We compared coercion data separately from both Wales and England with the WHO data for Great Britain. The restraint data that we considered for Germany were only from the region in south-west Germany that was considered previously.^11^ However, the WHO data included all of Germany.^9^

### Interventions examined

We considered involuntary hospital admission, seclusion and restraints in all included countries. The definitions and types of restraints for each country are delineated in the Supplementary Text and summarised below and in Table 1. All countries kept track of seclusions, but countries differed in both which restraint measures they reported and their definitions.

- Mechanical restraints, where mechanical devices such as belts or mittens are used to restrict movement: some countries (The Netherlands and New Zealand) considered bed rails or grids to be mechanical restraints; all countries except Wales reported some use of mechanical restraints (there was no use of mechanical restraint in Wales in the examined period). The US data combined mechanical and physical restraints without distinguishing the two, and we assumed the same proportions of restraints as those reported in another study^23^ to separate them.
- Physical restraints (also known as manual restraints), where a person's movements are restrained by one or more other persons without use of any device: only Australia, Germany, England, Wales and Ireland reported these separately from other restraints.
- Chemical restraint or involuntary medication involves the use of medication to restrain. It differs from therapeutic sedation in that it does not have a directly therapeutic purpose but is primarily employed to control undesirable behaviour.^25^ These are perhaps the most difficult restraints to monitor, because the use of restraints and involuntary medication may overlap, and some medication such as antipsychotics might be therapeutic in some cases but used as a restraint in others and might not be counted. England has a separate category for chemical restraints, and The Netherlands and Germany have separate categories for involuntary medication. We combined the two categories together.

Not every country reported on the same set of indicators. The only country that reported on all relevant types of coercion was England. The only intervention reported by all countries was seclusion. Germany did not report involuntary admissions in its country data-set or in the WHO Atlas. Only Germany and The Netherlands reported their data in approximately the same format.

We reported the different types of restraints separately when possible. We also combined the different types of restraints, including seclusion, to get an overall measure of RPI unless the overall measure was already reported (as delineated in Table 1, we combined the types for Australia, England, New Zealand and the USA).

We considered involuntary hospital admission separately; it was not considered an RPI in our calculations as it is a violation of autonomy of a different character compared with the other practices described in this paper. Although hospital admission durations are not a measure of coercion, we report them to give context.

### Years covered and reporting frequency

The years covered in this report include 2013 to 2020. All countries except The Netherlands had individual country profiles in the 2020 WHO Mental Health Atlas. The Netherlands’ most recent nationwide report was for 2014, although a recent study,^26^ together with updated general data gathered from mental health inspectorate sources such as the Dutch National Institute for Public Health and the Environment, suggested that the 2014 data continued to reflect current daily practice, warranting inclusion in the current study. Several other countries reported on different aspects of mental healthcare in 2020 compared with 2017. We used the 2020 results for all categories unless they were not available, in which case we used the most recent results, as indicated in the Supplementary Spreadsheet titled ‘Sheet 1. Final results for comparison and Sheet 2. WHO reports’.

Most countries reported on yearly numbers of coercive events or rates per population, or on monthly numbers that could be extrapolated to years by multiplying by 12. However, Japan only reported the numbers of people subject to coercive practices and the numbers of people in hospital for a single day of the year. The USA reported hours of restraint or seclusion per 1000 h of in-patient care, as well as the numerators and denominators.

### Rate calculation

In their 2010 systematic review, Steinert et al^8^ reported the percentage of admissions or patients exposed to RPI, as well as the mean duration, mean number of measures per patient, measures per 100 000 inhabitants per year and number of admissions. They pointed out that the measures per 100 000 inhabitants could most easily be used to compare countries. We followed that guidance by reporting on the numbers of each type of coercive measure per year per 100 000 population. The Japanese and US data required assumptions to retrieve rates per year. We therefore included other measures for total RPI that would allow for fewer assumptions for those countries’ data. Table 1 indicates which values each country reported and what extra material was used to make the conversion. The Supplementary Text and Spreadsheets include details of the calculations.

For each country, we attempted to quantify involuntary admissions and use of each type of restraint in 1 year in terms of:

- events per 100 000 population;
- patients affected by an event per 100 000 population;
- average number of events per affected patient;
- mean and median durations.

Additional comparisons made were:

1. patients affected by RPI per 1000 occupied bed-days;
2. number of RPI in 1 day per 1 000 000 population;
3. hours of RPI per 1000 h of patient care.

For most countries, we calculated the number of RPI event types per 100 000 population by dividing the absolute numbers in the year reported by the population in question. For Japan, we multiplied the number of people in restraints or seclusion on a given day (30 June) by 365 and divided that number by the average duration in days and by the population/100 000 to get the number of restraints or seclusions per 100 000 population per year. To compare the other countries with Japan more directly, we converted the values of RPI per year per 100 000 population to numbers of events per day per 1 000 000 population by dividing the numbers per year by 36.5. The US hours of restraint combined both mechanical and physical restraints, and we estimated their separate incidences using another study that determined durations and percentages of different restraints following injurious assaults.^23^ We then calculated the number of restraints by dividing the hours of restraint by the restraint durations. To calculate the hours of restraint per hours of patient care for the other countries, we calculated the hours of restraint by multiplying the restraint durations by the number of restraints, and we calculated the number of hours of patient care by multiplying bed-days by 24.

Physical interventions often have a skew in duration: many are short and fewer are longer,^18,23,27^ so mean values are larger than medians. For example, the mean duration of mechanical restraints in Japan for the years 2014 and 2019 was 30.5 days, whereas the median was 2 days.^18^ Medians are more representative of central tendency than means for skewed data such as these. However, some countries report numbers of events, whereas others report hours of restraint use. To transform between these types of measures, the mean duration rather than the median is necessary, because the mean is equal to the sum of all the duration measurements divided by the number of measurements. Where available, we report both mean and median in the Supplementary Spreadsheet.

Numbers of admissions were the most difficult to find, and we considered them to be the least reliable value. Many countries reported numbers of bed-days or numbers of patients seen in the year rather than numbers of admissions (Table 1 and Supplementary Spreadsheet). For Japan, we used the distributions of the durations of stay as of 30 June 2017 to calculate an estimate of the admissions per year (Supplementary Text S1 and Supplementary Spreadsheet).

From the WHO data-set, we considered involuntary treatment only for those confined to a hospital. We used the percentage of involuntary hospital admissions along with numbers of admissions per year to calculate numbers of involuntary hospital admissions per 100 000 population per year. We decided only to discuss differences of at least a factor of 10 to ensure we did not overemphasise differences that may have been caused by reporting errors.

## Results

### Comparisons from the nine countries’ individual data-sets

#### Overall summary

Durations and rates were highly variable (Table 2 and the Supplementary Spreadsheet Sheet 1: Final results for comparison and Sheets 3–13). For instance, the following measures varied by a factor of more than 100 between countries: numbers of mechanical restraints, seclusions and physical restraints per 100 000 population, number of RPI per 1 million population per day, hours of RPI per 1000 h of patient care, and mean durations of seclusions and mechanical restraints. Mean admission durations also varied by a factor of >100. Japan had the highest incidences for all of these except for numbers of physical restraints, which Japan does not track. High ratios could represent either exceptionally high or exceptionally low values. The lowest values for these categories varied between New Zealand, Wales, Germany, Australia and the USA. To focus on the highest values, we examined the ratios of highest to median values. These ratios were lower, but still more than 10 for numbers of mechanical restraints; number of RPI per 1 million population per day; hours of RPI per 1000 h of patient care; and durations of seclusion, mechanical restraints and admissions.

**Table 2:** Values for restrictive practices, to two significant figures^a^

Country	Year of comparison	Total population (millions)	Number of seclusions in 1 year per 100K population	Number of mechanical restraints in 1 year per 100K population	Number of physical restraints in 1 year per 100K population	Involuntary medication events in 1 year per 100K population	Number of RPI in one year per 100K population	Involuntary admissions per 100K population including WHO and country data-sets	Percentage of involuntary admissions	Mean duration of seclusion (h)	Mean duration of mechanical restraints (h)	Mean duration of physical restraint (h)	Mean admission duration (days)	RPI per 1000 bed-days	Number of RPI per 1M population per day	Number of people affected by RPI per 100K population per year	Hours of RPI per 1000 h of patient care	Average number of RPI events per affected patient
Australia	**2016/2017**	**24.6**	**48**	**6**	**54**		110	**220**	43	**5.8**			13	18	3.0			
England	**2017−2018**	**55.62**	**17**	**1.9**	**110**	**16**	150	73	41				76	**8.9**	4.1	**18**		8.2
Germany	**2013**	**2.109**	**80**	**81**	**0.24**	**2.3**	**160**			**7**	**8.2**	**0.25**	**25**	**8.7**	**4.5**	**38**	2.7	4.3
Ireland	**2020**	**4.882**	**38**	**3.1**	**82**		**120**	**42**	12	**14**	23	0.1	**26**	**13**	**3.3**	**39**	3.7	3.1
Japan	**2017**	**126.86**	190	120			260	220	47	470	730		2100	83	**190**		83	
The Netherlands	**2013**	**16.79**	**96**	**22**		**14**	130	140	27	**49**	**54**		71	3.6	3.6	42	6.8	2.7
New Zealand	**2016/2017**	**4.69**	**54**	**0.72**			**54**	122	54	**22**	**1.1**		28	8.7	1.5	**21**	7.9	2.6
USA	**2017**	325.8	7.5	18	7.0		32			6.4	4	0.79		3.6	0.88		**0.59**	
Wales	**2013**	**3.1**	1.0	**0**	60		**61**	91	**26**	**1**		**0.1**	**47**	**3.7**	1.7	**19**	0.022	3.2
Median		17	48	6	57	14	120	122	41	10.6	15.6	0.18	37	8.7	3.3	29	3.7	3.2
Ratio of highest to lowest		150	190	170	460	6.8	8.1	5.2	4.7	430	670	7.9	160	23	220	2.3	3800	3.2
Ratio of highest to median		19	4.0	20	1.9	1.1	2.2	1.8	1.3	44	47	4.5	57	9.6	58	1.4	23	2.6

RPI, restrictive practice incidents; WHO, World Health Organization.

a. Bold entries are those that did not have extra assumptions applied, as described in Table 1.

#### RPI rates and duration

Rates of RPI per 100 000 population were less variable between countries than rates for individual restraint types, as was found previously for European countries.^11^ However, the rates of RPI on a single day and the hours of RPI per 1000 h of patient care varied by factors of >200 and >4500, respectively, with Japan again having the highest values.

For most countries, there were substantially fewer reported mechanical restraint events than seclusions. Exceptions were that relative numbers of mechanical restraint events and seclusion events were nearly equal in Germany in a year (Table 2) and in Japan on a single day (Supplementary Spreadsheet), whereas the USA had more mechanical restraints, although both seclusion and restraint rates were low. Wales does not use mechanical restraint.

In England and Wales, there were many more physical restraint events than seclusions (Table 2), and mechanical restraint is effectively banned in most settings. In Australia, those rates were about the same, and in The Netherlands there were few reported physical restraints but more mechanical restraints and particularly seclusions, which remains the coercion measure of choice in most Dutch settings.

The incidences of involuntary medication did not vary much for the three countries that reported them (Table 2).

Mean durations of events were short and similar for physical restraints (all between 0.1 and 0.79 h, or 6 and 47 min) (Table 2). This is in contrast to the wide variation seen for seclusion and mechanical restraint.

#### Admissions and involuntary hospital admission

Involuntary and total admissions did not vary as much as RPI, with a median of 41% involuntary admissions and 390 total admissions per 100 000 population. The WHO Atlas^9^ reported 10% of admissions to in-patient facilities as involuntary across all countries, with medians of 125.6 total admissions per 100 000 population for all countries and 318.4 for high-income countries, similar to our results.

### Results from multi-country comparisons by WHO and the OECD

Categories that we compared between the OECD^12^ and WHO^9^ (Supplementary Spreadsheet, Sheet 3: WHO *v.* OECD reports) indicated that OECD numbers varied by less than 30% of the WHO figures for suicide rates, government spending on mental health and the number of mental health nurses per 100 000 population. (The OECD report relied on the WHO Atlas for some of these figures.) However, WHO and OECD figures differed by up to 137% for the number of psychiatrists per 100 000 people (the largest difference was for Ireland, with eight according to WHO and 19 according to OECD reports) and 790% for the number of psychologists per 100 000 population (the largest differences were for Japan, with 28 according to the WHO and three for the OECD reports; and New Zealand, with nine for WHO and 86 for OECD).

The only measures in the WHO figures (Supplementary Spreadsheet and ref. ^9^) that varied by more than a factor of 100 among included countries were the amount of government funding of mental health as a proportion of total healthcare (which was affected most by the very low value of 0.05 in the USA), the number of visits to hospital-based out-patient facilities per 100 000 population (37 730 in Australia *v.* 251 in Japan) and the number of admissions to general hospital psychiatric unit beds per 100 000 population (987 for Germany *v.* 10 for the USA).

#### Comparison of country reports with WHO and OECD studies

A Health Policy study about the OECD asked countries’ representatives to report the number of restraints and seclusions in a year, and plotted them in its Figure 3.13.^12^ The values for Great Britain, Ireland and New Zealand's seclusion numbers and Great Britain's restraint use in these plots were close to our results (Supplementary Spreadsheet OECD_vs_WHO). However, the values for Japan were close to Japan's reported values for the single day of 30 June, suggesting a misunderstanding in the report. (Figure 3.13 in ref. ^12^ suggests that there were between 12 000 and 13 000 people in seclusion and 11 000 to 12 000 people restrained sometime in 2019 or the most closely available year, whereas the 630 (30 June) report for 2018^17^ gives values of 12 364 in seclusion and 11 362 in restraints in a single day.) No other countries in this study were represented in Figure 3.13 of the OECD report.^12^

There was a similar discrepancy for the involuntary hospital admission numbers for Japan in the WHO Atlas (Supplementary Spreadsheet). The WHO Atlas for Japan for 2017^9^ reports 125 287 involuntary hospital admissions for the year, whereas their government's data source for the single day of 30 June 2017 (the ‘630’ (30 June) report)^17^ indicates 133 450 people in hospital who were not voluntary patients.

## Discussion

### Main scientific findings and implications

This is the most comprehensive overview of rates of coercive practices between countries attempted to date. The primary findings are that although large variations in reporting methods and mechanisms make comparisons between countries difficult, we have devised valid methods to make some relevant comparisons and found 10- to 100-fold variations in the restraint rates of the selected high-income countries. It therefore seems unlikely that such marked differences are solely related to reporting differences. Moreover, it is not a strong concern that some countries report more types of restraints than others and are thus unfairly disadvantaged when reporting RPI: the country with the largest RPI per 100 000 population, Japan, only considers two types of RPI (seclusion and mechanical restraint), whereas the only country that reports on all types of RPI, England, has RPI rates of 150 per 100 000 population, close to the median of 120.

Our previous work has shown that rates of coercion also vary by orders of magnitude (factors of 10 or more) between regions and even hospitals within the same country.^10,11,28^ This suggests that individual hospitals create different cultures, or that different hospitals tend to see patients with varying degrees and types of illnesses, or both.^29^ Flammer et al^30^ show that in Germany, the degree of legal compulsion (a measure of need and disturbance) is the only factor that correlates with incidence of restraint. Reid and Price^27^ suggest there are factors associated with scale, such as aggregation of persons with more need and therefore more restraints, in larger hospitals. Countries could examine the variations between their hospitals to help them determine which practices are most useful to reduce restraints and could use those examples to help reduce restraints in other hospitals.

As both rates and durations of coercive measures are higher in Japan than in other countries, we hypothesise that some countries’ tolerance of one type of coercion may extend to other types and to durations (Table 2). However, the fact that countries’ restraint rates differ by a factor of over 100 for several individual types of restraints but by a factor of less than 10 when considering total RPI suggests that in many countries, certain types of coercive measures are favoured, and the favoured method may vary between countries, as previously suggested.^11,29^ The high variation in restraint rates between countries despite limited variation in most measures reported in the WHO Atlas suggests that the tradition and culture of the country may be important. For example, Japanese psychiatrists justify involuntary admission, arguing that people in a psychiatric crisis cannot decide or understand and need protection.^31^

There may be other countries that could be included in the future in a wider study using these same methods. Correlations between different restraint measures and variables in the WHO Atlas should be examined for data-sets of larger numbers of countries when and if they become available and standardised. Individual countries should improve the reporting of RPI by mental health providers and publish them in a systematic manner as we discuss below.

### Limitations

#### Under- or overreporting

Reporting issues may occur in all jurisdictions and are nearly impossible to quantify, and they will be nearly as much of a concern for any study that tries to compare restraints in different hospitals or regions within a single country as for this study.

- Reporting can bring criticism,^32^ creating incentives not to report. As noted above, definitions can be variable. In addition, some forms of restraint may not be recognised as restraint by those doing them. Examples include improvised use of furniture to ‘pen in’ a frail elderly person, which could meet mechanical restraint definitions in most jurisdictions but might not be seen as such by the people doing it (personal experience of authors).
- There may be a lack of a framework for reporting restraints. In the UK in 2013, several providers did not provide figures on restraint in response to a legally mandated request from a major mental health charity.^32^ Some staff members may feel that physical restraint, for example, need not be reported in addition to an enforced injection or mechanical restraints, because it is obvious to them that physical restraint would have occurred to facilitate the injection.^8^
- Some people are affected by several different types of restraint at the same time, and they may contribute separately to the RPI rates. Moreover, people may undergo multiple episodes of RPI. Therefore, there will always be fewer people affected by RPI than the numbers of RPI events.
- Events can be recorded as happening at the time they started, at the time they are reported or not at all, because the task to report is handed on to a colleague and not executed.
- Events may unfold chaotically and the details may be forgotten before there is time to record them. Reporting mechanisms that are not electronic may mean that reports are lost before they are recorded in a database.
- Finally, some restraint episodes have phases that move between physical and mechanical restraint and seclusion, and back to physical, and some jurisdictions do not require that the repeated elements of the same nature are reported, nor can all providers easily record this in their forms.

#### Problems in databases

The main periods covered by the two previous studies differed by 4 years, and when we attempted to examine or re-examine the countries’ online data-sets, many of them had been updated and revised, with data for past years often no longer available online. Australia,^13^ Japan^17^ and the USA^22^ keep data for a number of years available online, with minor updates. England has years of monthly data, available freely, albeit incompletely returned by providers. There, legislation mandates the need for mental health units to report along well-defined lines (Seni's Law). This augments previously existing general Freedom of Information rules requiring public bodies to report data on request for a fee, which did not lead to complete reporting in 2013.^27^ Ireland has only the current year's data available freely, whereas south-west Germany, The Netherlands, New Zealand and Wales require special access to get current data. We have copies of the original data-sets that we will share with interested researchers on request, but government entities may charge investigators to verify them independently.

#### Problems in calculations

Our biggest assumptions in calculations were for admissions and involuntary hospital admissions, particularly for New Zealand, and for the numbers of seclusions and restraints for the USA (Supplementary Spreadsheet, Supplementary Text and Table 1).

For both Japan and the USA, we converted to numbers of events per year based on estimates of the durations of restraint and seclusion events from other studies,^18,23^ but these extrapolations meant that the numbers of events per year were not as robust as those for the other countries. There is debate in the literature regarding the average length of restraints in Japan. The official report that we used reports a mean of 30 days and median of 2 days.^18^ This was based on a survey that had a 19% response rate. Another study that examined detailed logs from 11 hospitals found a median of 19 days and mean of 96.2 days for mechanical restraint.^33^ We therefore also attempted to convert the other countries’ units to those of Japan and the USA as discussed above, with some success.

#### Limited generalisability to low- and middle-income countries

All data on coercive measures that we were able to access in detail were from high-income countries. Limited data are available in the literature from low- and middle-income countries, and those that are available are from individual hospitals. For example, in one hospital in Mysuru, South India, 67% of in-patients were subjected to some form of RPI and 75% were admitted involuntarily,^34^ compared with the WHO average of 10% for involuntary admissions and the median of 41% in this study.

### Implications for clinical practice

Research shows that coercive practices are psychologically and physically harmful, with harms including death,^35,36^ and that alternatives to coercive practices are cost-effective, lead to fewer injuries to staff and patients, and result in quicker resolution of crises;^37^ however, coercive practices are still used at high rates. This suggests that stronger efforts are required to provide alternatives to coercion and hence to reduce coercion rates.

There is a growing body of literature on methods to reduce coercion, including papers in this issue. A review by Hirsch et al^38^ focused on what methods work best to reduce coercion. They determined that sustained successes were achieved when a variety of measures were maintained over a long period of time, driven at a high management level,^38^ such as recommended in the Six Core Strategies.^6^ The huge differences between some countries and the successes of some sustained strategies show that reducing coercion is possible.

### Policy implications

Although waiting for the availability of standardised measures may be ideal, it may unnecessarily delay efforts to benchmark examples of existing good practice. Just as countries use international educational attainment reports to determine whether to change their educational practices, they could use these comparisons of available mental health indicators to spur changes in training, laws or recommended practice. Improving the standardised reporting of RPI, developing and implementing evidence-based practice, supporting policies that reduce RPI, and coordinating global efforts between the WPA and the WHO should all help to reduce restraint worldwide.

In addition to the data suggested by Steinert et al,^8^ we expand to all coercive measures the following suggestions from Newton-Howes et al,^10^ which were initially for mechanical restraints, as a minimum set of measures to monitor, and we urge the WHO to consider adding these to the questions in their Atlas.

- Number of episodes of involuntary hospital admissions per year, and number of unique people admitted to hospital involuntarily during each year;
- Separate reporting for mechanical, physical and chemical restraints and seclusion, including the absolute numbers of people affected, how many times they were restrained or secluded and for how long, counts of incidents and people affected per year per population, and statistics on duration (e.g. mean, median and 75th, 90th and 95th percentiles) for each type of practice. Duration statistics are particularly important for mechanical restraint, where risk of death increases with duration of use.^39^
- Keeping track of restrictive practices is not enough; each nation should continually work towards reducing their numbers. The metrics should be used to measure the effectiveness of coercion reduction techniques. In particular, the sustainability of any intervention should be monitored.

### How to implement practice monitoring

We consider that the best way to keep track of coercion is to have each episode of coercion treated as a safety incident and recorded as it occurs, in terms of who was subjected to the coercion, what type was used, and when it started and ended, with the data recorded in a spreadsheet or database. This should not be much more burdensome than the current practice of keeping logs of ward activity. The digital data could be anonymised and simple calculations made to get the statistics discussed above. As suggested in the Six Core Strategies,^6^ after every episode of coercion, there should be a group meeting of the people involved, including the patients, their formal or informal caregivers, and the nurses and psychiatrists, to determine what could be done better in future to prevent the need for coercion.

Our findings that physical (i.e. manual) restraints had the shortest durations suggest that one way to reduce overly long durations is to require continuous one-on-one monitoring of any person assigned to isolation or restraint of any kind. For safety reasons, aspects of this already occur in some jurisdictions (e.g. the UK's Mental Health Act Code of Practice states: ‘*26.80 An individual who is mechanically restrained should remain under continuous observation throughout*’^40^). The extra staff required should help to make the procedure safer and would also provide a financial incentive in the form of lower personnel costs for hospitals that successfully implement alternatives to coercion.

We urge the WPA and WHO to recommend and publicise the measures we suggest for keeping track of RPI. The WHO could help to spur other countries to begin keeping track of RPI by including questions about them in their future Mental Health Atlases.

## Supporting information

Savage et al. supplementary material 1 Savage et al. supplementary material

Savage et al. supplementary material 2 Savage et al. supplementary material

## Data Availability

All calculations are available in the Supplementary Material, which also gives links to the data sources used in the study, or from the corresponding author (M.K.S.).

## Supplementary material

Supplementary material, including spreadsheets and text with detailed information on the calculations and practices of each country, is available online at: https://doi.org/10.1192/bjo.2023.613.
