# Supplementary material for: Comparison of coercive practices in worldwide mental healthcare: overcoming difficulties resulting from variations in monitoring strategies
Source: BJPsych Open. 2024 Jan 11;10(1):e26. doi: 10.1192/bjo.2023.613 (PMC10790218; doi:10.1192/bjo.2023.613)
Supplement: Savage et al. supplementary material 1 — Savage et al. supplementary material [file S2056472423006130sup001.doc]

Title: Comparison of coercive practices in worldwide mental health care: Overcoming difficulties from variations in monitoring strategies

Prepared for an invited supplement to the *British Journal of Psychiatry Open*

Authors: Martha K. Savage, Peter Lepping, Giles Newton-Howes, Richard Arnold, Vincent S. Staggs, Steven Kisely, Toshio Hasegawa, Keith S. Reid, Eric O. Noorthoorn.

**Supplementary Text S1: Specific details about mental health policies, data sources, preparation and analysis for separate nations, OECD and WHO reports.**

*Australia.*

For Australia we used the Australian Restrictive Practices Tables (1) published by the government agency “Australian Institute of Health and Welfare”. These tables consider all public sector acute specialised mental health hospital services. Australia’s private hospitals do not treat patients who are compulsorily hospitalised, and it is thought that they are unlikely to use restraints much since their patients are free to leave and are paying with their own (or their insurance’s) money. Forensic patients were included in a limited number of cases, and all ages were included. About 10% of the discharges from specialist psychiatric care facilities in Australia were for people older than 65, and that percentage was stable between 2015 and 2018.

Australia reports involuntary mental health care, seclusion and physical and mechanical restraint. The Australia Institute of Health and Welfare (2) defines seclusion as “the confinement of the consumer/patient at any time of the day or night alone in a room or area from which free exit is prevented”. Mechanical restraint is defined as “the application of devices (including belts, harnesses, manacles, sheets and straps) on a person’s body to restrict his or her movement”, and physical restraint is defined as “the application by health care staff of hands-on immobilisation or the physical restriction of a person to prevent the person from harming himself/herself or endangering others or to ensure the provision of essential medical treatment”. In its “restrictive practices tables”(1), it reports the numbers of admissions and involuntary separations (which is approximately the same as the number of involuntary admissions). They report seclusions per 1000-bed days and average seclusion duration in hours, the proportion of admitted care episodes that have a seclusion event, and the average number of seclusion events per episode with seclusion. They also keep track of the absolute numbers of the restraint and seclusion events and numbers of admissions, so we were able to use those absolute numbers and the total population to give rates per 100,000 population as well as the published numbers per 1000-bed days. They do not include durations of physical or mechanical restraint or duration of admissions, or unique people secluded or restrained.

*England*

For England we used the England Mental Health Bulletin report for 2017/2018 (3). The report includes people who were in contact with the secondary mental health, disabilities and autism services in England through the National Health Service in the year 2017-18. We used the results for the “adult mental health cluster, ISB 1509”. For the number of people we added up the totals from “Table 3.1: Number of Adult Mental Health Care Clusters assigned at the end of the year by gender, age group and super class, 2017-18” of all ages from under 18 to 90 or over, for all genders for the categories of Non Psychotic, Psychotic, and Organic.

England’s tables have categories of “Physical restraint-Prone, Physical restraint-Excluding prone, Chemical restraint, Mechanical restraint, Seclusion and Segregation” for both number of people subject to a restrictive intervention and for number of restrictive interventions (3). The definitions are on the internet at this location as of 5 March 2023: (https://digital.nhs.uk/data-and-information/data-collections-and-data-sets/data-collections/assuring-transformation/definitions-for-restrictive-interventions-for-use-in-assuring-transformation). Physical restraint is “any direct physical contact where the intervener’s intention is to prevent, restrict, or subdue movement of the body, or part of the body of another” and Prone refers to “A physical restraint in a chest down position, regardless of whether the person’s face is down or to the side.” Mechanical restraint is “the enforced use of mechanical aids such as belts, cuffs and restraints to forcibly control a patient’s movement for the prime purpose of behavioural control.” Chemical restraint is “the use of medication which is prescribed, and administered (whether orally or by injection) for the purpose of controlling or subduing disturbed/violent behaviour, where it is not prescribed for the treatment of a formally identified physical or mental illness”. Seclusion is “the supervised confinement and isolation of a patient away from other patients, in an area from which the patient is prevented from leaving, where it is of immediate necessity for the purpose of the containment of severe behavioural disturbance which is likely to cause harm to others.” Long term segregation refers to a situation where: ”in order to reduce a sustained risk of harm posed by the patient to others, which is a constant feature of their presentation, a patient is not allowed to mix freely with other patients on the ward/unit on a long term basis”. We obtained the numbers of RPI events and of people subject to RPI from their table 7.1. The total number of people restrained is less than the sum of each category, because some people will undergo more than one type of intervention and some episodes will include more than one type of intervention. We used the smaller number in the comparison to other countries. The total number of RPI events in their table was larger than the totals from adding each type of intervention, so we again used the smaller number. Also, to make the figures comparable to other studies, we combined the numbers in “Physical restraint-Prone and Physical restraint-Excluding prone to get numbers subject to Physical Restraint. We also added the numbers for “Seclusion” and “Long Term Segregation” to get the total number of people in seclusion.

There were no reports of durations of any of the restrictive measures. We report the number of admissions from their sheet 5.1 “Admissions, discharges and average daily occupied beds by year, 2011-12 to 2017-18”, column “MHSDS 2017-2018”. The number of bed days comes from sheet 4.1 “Number of in-year bed days by gender, type of provider and age band, 2017-18”. We include the total for all genders and all ages. We calculate the mean admission duration by dividing the number of bed-days by the number of admissions. There was no information about numbers of involuntary admissions, so we use the WHO Atlas (4) for the percentage of involuntary admissions to calculate the total number of involuntary admissions. Table 1.1 gives us the total number of people in hospital from the “total admitted”.

*Southwest Germany*

For Southwest Germany we used the database used by Lepping (5) for 2013, from the Centre for Psychiatry Südwürttemberg and its affiliates. They provide inpatient psychiatric care for about two million inhabitants of nine counties in Southwest Germany, both voluntary patients and those detained under mental health legislation. For six counties, the centre is the only provider and in two counties there are also university hospitals providing inpatient care. For one county, the Centre only provides inpatient care for children and

adolescents.

As outlined by Lepping (5), Southwest Germany reports seclusion, mechanical restraint and involuntary medication. Seclusion is defined as bringing the patient into a locked room where he/she is alone and able to move freely but unable to leave due to a locked door. Mechanical restraint refers to the use of belts, bed grids or protection blankets to fix the patient to the bed. According to internal hospital policies, patients have to be constantly and personally monitored during mechanical restraint. Involuntary medication is defined as application of medication by force or by definite psychological pressure. The database includes exact data on the duration of each measure, collected according to clear definitions. The data on coercive measures can be considered highly accurate due to the legal obligations of documentation. We also include the total number of admissions and the population of the region as published in Lepping et al. (5). Germany does not report the number of involuntary admissions, either here or in the WHO Atlas (4).

*Ireland:*

We used the restrictive practices report for 2020 (6). This report includes all approved mental health facilities. These include one forensic facility as well as one centre for learning disability care. An approved centre is defined in the report as a hospital or other inpatient facility for the care and treatment of persons suffering from mental illness or mental disorder which is registered pursuant to the Irish Mental Health Act.

Ireland’s report (6) included mechanical restraint, physical restraint and seclusion. It reported numbers of events and numbers of people suffering the events and total duration of the events in the year. We divided the total duration of the events by the number of the events to get the average duration per event. We obtained the number of hospital admissions, bed-days and involuntary hospitalisations from a separate report (7). Mechanical restraint is defined as “the use of devices or bodily garments for the purpose of preventing or limiting the free movement of a patient’s body”. The use of bed rails or cot sides is excluded from the definition. Physical restraint is “the use of physical force (by one or more persons) for the purpose of preventing the free movement of a resident’s body when he or she poses an immediate threat of serious harm to self or others”. This may be done to manage aggression or to administer involuntary medication. Seclusion is “the placing or leaving of a person in any room alone, at any time, day or night, with the exit door locked or fastened or held in such a way as to prevent the person from leaving”. If a patient needs physical restraint in order to be secluded, both events are counted.

*Japan*

For Japan we used the yearly “630 report” (8), which reports a survey carried out on all psychiatric wards on 30 June every year. Most of Japan’s dementia care patients are not in the psychiatric hospital system, but if they exhibit concerning symptoms they may be placed in a psychiatric hospital. Earlier we considered different age groups to try to remove dementia patients (9). For this paper instead we include all ages to be consistent with the other studies.

Each year Japan surveys the numbers of people on 30 June in hospital, under compulsory admission orders, seclusion and restraint orders, and distributions of how long each person has been in the hospital up to that day. Seclusion and restraint orders allow inpatients to be secluded by the nurses on that day. They could be used more than once, or not at all, on a given day (10, 11). No data is provided for physical restraints or restraint duration in the yearly surveys. However, a report published by Yamanouchi et al for the Japanese Ministry for Health, Labor and Welfare on 30 March 2021 (10) determined, for selected hospitals for two years (2014 and 2019), how many patients were actually restrained when they had a restraint order, and it gives the distribution of lengths of restraint and seclusion, as well as the mean and median, in days (Their Figures 18 and 19). We averaged the two years’ results to find a mean of 30.5 and 19.5 and medians of 2 and 3.5 for mechanical restraint and seclusion, respectively. 96.9% of patients with restraint orders were actually restrained in 2019 compared to 96.5% in 2014 (10). This official report (10) was based on a survey that had only a 19% return rate. Another study using a smaller number of hospitals that cooperated with the Japanese Hospital and Community Psychiatry Association, found a mean duration of 96 days (12).

The number of voluntary and involuntary inpatients on 30 June are reported, and the distributions of the length of time all people had been in hospital before 30 June are reported in groups of <1 month, 1-3 months, 3-6 months, 6-12 months, 1-5 years, 5-10 years, 10-20 years and >20 years (Supplementary Spreadsheet Table 13 Japan_duration_hospital_stay). We consider the number of involuntary inpatients to be the total of the numbers of patients in the “involuntary” and “medical protection” categories. Their “involuntary” category is for patients determined to be a danger to themselves or others by two psychiatrists who independently evaluate the patient, while their “medical protection” category is for patients determined to need care by one psychiatrist and a guardian, usually a family member (13). We do not consider “other” or “unclear”, because those are each less than 1% of the total inpatients. The median length of stay up to the reporting date was 1-5 years. We used these distributions to estimate the mean duration, using the midpoints of the distributions weighted by the proportion of the population that was in that category. For >20 years there is no midpoint, so we used a value of 300 months, or 25 years, which we consider conservative. That population makes up 9% of the total population of admitted people. We calculate that the mean length of stay for patients to 30 June is 69.44 months, or 2112 days. The minimum mean length of stay for all patients is then 2112 days. This is a minimum because most patients will continue in hospital past the measurement day. If we assume that on average, a person will stay just as long in the future as they have up to the present, then the mean length of stay will be 4224 days.

We use the same data to estimate the number of admissions by calculating the proportion of admissions that each length group could represent. We consider that the number of admissions represented by people in a group would be the midpoint of the number of months that group had been in divided by 12. For example, those in for < 1 month average 0.5 months, and could be replaced 23 times by other people who were also in for one month. The total number of admissions represented by the < 1 month group is then 24 times the number of people in that admission group. Using this approach, we estimate that the number of admissions is 1,063,204 in one year. If the average length of stay is twice as long as their stay up to present, or 4224 days, then the number of admissions in one year is halved, to 531,602, yielding 413 admissions per 100,000 population.

*Netherlands*

For the Netherlands, data is from the same 2013 database (14) that was used in (5) and (15). The database represented an estimated national coverage of 95% of all admissions and 98% of all coercive measures. It includes figures from child psychiatry facilities and facilities for the elderly, but forensic patients and specialist LD services were not included (16). The accuracy of the data reporting was very good when it was formally tested (17).

The Netherlands’ database included seclusion, mechanical and physical restraint, involuntary medication, and involuntary feeding. However, we did not include the involuntary feeding data because it is the only country that includes such data. We obtained the total number of admissions and the population of the region from the same source used in Lepping et al. (5). The rooms used for seclusion preclude mechanical restraints, so that the two populations are distinct. However, 13% of the people who are in seclusion are undergoing chemical restraint at the same time. Seclusion is defined as bringing the patient into a locked room where he/she is alone and able to move freely but unable to leave due to a locked door. Mechanical restraint refers to the use of belts, bed grids or protection blankets to fix the patient to the bed. Physical restraint refers to immobilizing the patient by means of physical force. Involuntary medication is defined as application of medication by force where the patient shows overt resistance.

*New Zealand*

For New Zealand in 2016-2017, we use the database that we created through Official Information Act requests to each hospital in the country for mechanical restraints in Newton-Howes et al. (9), and for seclusion we use New Zealand’s yearly report from the Ministry of Health on Mental Health and Addiction services, using the tables for the inpatient mental health services(18), which include all public hospitals. There are few private hospitals and those do not use restraints. Seclusion is defined in these tables as “The placing of a client, at any time and for any duration, alone in a room or area from which they cannot freely exit.” The tables do not include dementia wards or learning disabled, or forensic wards. Here we only consider the aggregated results for all clients, without considering the age, sex or ethnicity.

The types of mechanical restraints used in 2016-2017 were two incidences of bed rails as well as wrist and leg restraints, and belts that go across a lap whilst sitting in a chair. The Office of Mental Health (18) reports on key performance indicators for the 2016/2017 year for total number of seclusion events in the year (their Table 46), and clients of any age admitted and bed-nights seen by the in-patient team (their Table 21). The key performance indicators included total seclusion hours per person secluded, average duration of seclusion events in hours, seclusion hours per 100,000 population, number of seclusion events per person secluded, number of seclusion events per 100K population, and unique people secluded per 100 k population. There was no table heading for admissions, but there was a table for the numbers and purposes of “Health of the Nation Outcome Scales” (HoNOS) given. Every time somebody is admitted, they are given an HoNOS test, so the numbers of these tests given for the purpose of admissions should equal the number of total admissions. This will include the number of people who were admitted more than once. We do not know the total number of people who were mechanically restrained, but it cannot be larger than the number of restraint episodes, which is only 34. Therefore to get an estimate of people subjected to RPI in the year we add the number of people subjected to seclusion (960) to the number of restraint episodes, with a realisation that it could be slightly high.

To estimate involuntary hospitalisation also required understanding the path of the Mental Health Act. In New Zealand, if it is considered that a person may be a threat to themselves or others, they are first put under compulsory assessment up to two weeks under “section 11” to determine whether they should be put under compulsory treatment through the Mental Health Act. Almost all people will be in the hospital during their evaluation period. There are a few more steps, culminating in about 74% of people under assessment being put under the Mental Health Act. We consider the number of people in the year who were under section 11 to be equal to the number of compulsory patients, since almost everybody progresses first through section 11 before going through any other steps toward compulsory treatment. Some people could be put under section 11 more than once in a year. To get the involuntary admissions, we assume the percentage of re-admitted patients is the same in the compulsory inpatients as in the rest of the inpatients.

*United States*

For the US: the Centers for Medicare and Medicaid Services (CMS) Hospital Compare database, (19, 20) was used for 2017. The CMS provides measures of quality designed to help people decide which hospitals to use. The database included the majority of both public and private psychiatric hospitals, general hospitals, and VA medical centres that provided 24-hour inpatient care, but because not all such facilities provided restraint data to CMS, our count of restraints is likely an under-estimate. For most years, including 2017, data on patient ages were not available. For the available years, 2013, 2014 and 2018, the population >65 years of age made up about 17% of the bed-days. People younger than 18 made up a negligible proportion of bed-days in hospital, and also of restrained people.

The CMS dataset (19, 20) reported annual hours of patient seclusion; annual hours of patient restraint, including mechanical and physical restraint, which are not tracked separately; and annual patient days. Physical restraint is defined by CMS as “…any manual method or physical or mechanical device, material, or equipment that immobilizes or reduces the ability of a patient to move his or her arms, legs, body, or head freely when it is used as a restriction to manage a patient’s behaviour or restrict the patient’s freedom of movement and is not a standard treatment for the patient’s medical or psychiatric condition.” Seclusion is defined as “the involuntary confinement of a patient alone in a room or an area where the patient is physically prevented from leaving. This includes but is not limited to¹: Manually or electronically locked doors, one-way doors, the presence of staff proximal to the room preventing exit or the threat of consequences if the patient leaves the room (¹ 42 CFR Part 482, Medicare and Medicaid Programs; Hospital Conditions of Participation: Patient's Rights” from [https://manual.jointcommission.org/releases/TJC2018A/DataElem0149.html downloded 5 March 2023](https://manual.jointcommission.org/releases/TJC2018A/DataElem0149.html%20downloded%205%20March%202023)). These data elements are used to compute hours of seclusion per 1,000 inpatient hours and hours of restraint per 1,000 inpatient hours. To translate from number of hours of restraint or seclusion to numbers of restraint or seclusion events, we estimated the average proportion of physical and mechanical restraints and the number of hours of restraint and seclusion per event using data on duration of seclusion and restraint in response to injurious assaults (21). We calculated the average duration of restraint per event as 4.06 hours for mechanical restraint, 0.79 hours for physical (manual) restraint and 6.4 for seclusion. We attempted to find the numbers of voluntary and involuntary hospital admissions from a report for 2014 about inpatient capacity (22), but that report was not able to calculate admissions except at community hospitals, which most likely left out the state hospitals with the most severe illnesses. Therefore we do not report numbers or durations of admissions or involuntary admissions.

*Wales*

For Wales, we used the 2013 data collected through Official Information Act requests and published by Lepping et al. (2016) excluding the Learning Disabled services. Forensic services are very limited and were not separable and so are included in the values used.

The information we have for Wales is for total RPI events and the percentage of seclusion events (5). RPI is defined in Wales as “direct physical contact between persons where reasonable force is positively applied against resistance, either to restrict movement or mobility or to disengage from harmful behaviour displayed by an individual” (Welsh Assembly Government Framework for restrictive physical intervention 2005). Mechanical restraint, whereby a device is used to restrain a person, is banned there. Likewise, chemical restraint is not recorded, but about half of physical restraint incidents are associated with (mostly voluntary) medication use. The data therefore cover mainly physical restraint and seclusion, and we calculate the physical restraint as the number of RPI events minus the number of seclusions. The percentage of involuntary patients of 26% was taken from the 2020/2021 data (23), as the 2013 data was no longer available.

*World Health Organisation (WHO) Mental Health Atlas (4)*

The WHO Mental Health Atlas is prepared every three years, with the most recent being for 2020. Its goal is to provide worldwide information about mental health services and resources, including policies, legislation and financing and the availability and utilisation of services, and to track changes over time in relation to previous goals set. Questionnaires are sent to a single point contact in each country, and not all questions are answered by every country. 171 of the 194 member countries of WHO answered at least some of the questions. We considered the following categories that might be relevant to the coercive practices, and they are reported in the supplemental spreadsheets in the tab “WHO reports”, and in tab “WHO vs OECD reports” in columns C, E, F, G, J, K, M, N, P, S.

The following columns are defined in the WHO reports spreadsheet, which were each taken from the individual country report sheets:

Definitions of types of care facilities in the WHO report (Appendix B):

**“Mental hospital:** A specialized hospital-based facility that provides inpatient care and long-stay residential services for people with mental health conditions. Other names include mental health hospital and psychiatric hospital. Includes: Public and private non-profit and for-profit facilities; forensic inpatient facilities; mental hospitals for children and adolescents and other specific groups (e.g. older adults). Excludes: Community-based psychiatric inpatient units; facilities that treat only people with alcohol and substance use problems or intellectual disability; psychiatric units in general hospitals; and mental health community residential facilities.

**“Psychiatric unit in a general hospital:** A psychiatric unit that provides inpatient care within a community-based hospital facility (e.g. general hospital); the period of stay is usually short (weeks to months) and the hospital also provides services related to other medical specialties. **Includes:** Public and private non-profit and for-profit facilities; psychiatric wards or units in general hospitals, including those for children and adolescents or other specific groups (e.g. older adults). **Excludes:** Mental hospitals; community residential facilities; facilities for alcohol and substance use problems or intellectual disability only.

**“Mental health community residential facility:** A non-hospital, community-based mental health facility providing overnight residence for people with mental health conditions. Both public and private non-profit and for-profit facilities are included. **Includes:** Staffed or unstaffed group homes or hostels for people with mental health conditions; halfway houses; therapeutic communities. **Excludes:** mental hospitals; facilities for alcohol and substance use problems or intellectual disability only; residential facilities for older adults; institutions

treating neurological disorders or physical disability problems.

**“Mental health day treatment facility:** A facility providing care and activities for groups of users during the day that last for half a day or one full day (including those for children and adolescents only or other specific groups, e.g. older adults). **Includes:** Day or daycare centres; sheltered workshops; club houses; drop-in centres. Both public and private non-profit and for-profit facilities are included. **Excludes:** Day treatment facilities for inpatients; facilities for alcohol and substance use problems or intellectual disability only.

**“Mental health outpatient facility:** An outpatient facility that manages mental health conditions and related clinical and social problems. **Includes:** Community mental health centres; mental health outpatient clinics or departments in general or mental hospitals

(including those for specific mental health conditions, treatments or user groups, e.g. older adults). Both public and private non-profit and for-profit facilities are included. **Excludes:** Private practice; facilities for alcohol and substance use problems or intellectual disability only.

**“Other residential facility:** A residential facility that houses people with mental health conditions but does not meet the definition for community residential facility or any other defined mental health facility. **Includes:** Residential facilities specifically for people

with intellectual disability, for people with substance use problems or for people with dementia; residential facilities that formally are not mental health facilities but where most

residents have diagnosable mental health conditions.

**Year of report:** The year named on the WHO Atlas report. 2020 was published in 2021 for data collected in 2020, etc.

**Population:** self-explanatory

**Income Group:** High-income countries are those that were designated high-income by the World Bank. All countries here were considered high-income, which in 2020 meant that the gross national income per capita was US$ 12,376 or more.

**Mental Health expenditure per person, Govt expenditure on mental health as percent of health expenditure, and Govt expenditure on mental hospitals as % of mental health expenditure:** the Questionnaire asked for “Health expenditure and percentages attributed to specific types of expenditure, including government expenditures on mental hospitals, mental health care in general hospitals, mental health prevention and promotion, community mental health services and mental health supports at primary health care level”. Each country report was in their own currency, and we converted them into US dollars and EURO using the exchange rate on 25 April 2022.

**WHO region:** WHO breaks the world into several regions; those represented here are EURO for Europe, WPRO for Western Pacific, and AMRO for America.

**Age-standardised suicide rate per 100K**: “Data on age-standardized suicide rates per 100,000 population were taken from the WHO Global Health Observatory”(24)

**Policy/legislation in line with human rights convention:** Countries were asked to report how well their policies aligned with the human rights convention. The WHO Atlas notes that “However, we know that policies, plans and laws do not fully align with international human rights standards. Apart from the fact that they allow involuntary admission and treatment, and seclusion and restraint, most policies, plans or laws do not promote supported decision-making”. The responses by the countries here are given as n/m, where n is a scale from 1-5 for whether their policies and plans are in line with human rights instruments, and m is a scale from 1-5 that indicates whether their laws are in compliance with human rights instruments. Most countries answered 5/5, indicating that both their policies and laws were in compliance with the following: 1) Policy/plan promotes transition towards mental health services based in the community (including mental health care integrated into general hospitals and primary care); 2) Policy/plan pays explicit attention to respect of the rights of people with mental health conditions and psychosocial disabilities as well as at-risk populations; 3) Policy/plan promotes a full range of services and supports to enable people to live independently and be included in the community (including rehabilitation services, social services, educational, vocational and employment opportunities, housing services and supports, etc.); 4) Policy/plan promotes a recovery approach to mental health care, which emphasizes support for individuals to achieve their aspirations and goals, with mental health service users driving the development of their treatment and recovery plans; 5) Policy/plan promotes the participation of persons with mental health conditions and psychosocial disabilities in decision-making processes about issues affecting them (e.g. policies, laws, service reform, service delivery). (5 = fully in line).

Law compliance with human rights instruments self-rated 5 points checklist items: 1) Law promotes transition towards community-based mental health services (including mental health integrated into general hospitals and primary care); 2) Law promotes the rights of people with mental health conditions and psychosocial disabilities to exercise their legal capacity; 3) Law promotes alternatives to coercive practice; 4) Law provides for procedures to enable people with mental health conditions and psychosocial disabilities to protect their rights and file appeals and complaints to an independent legal body; 5) Law provides for

regular inspections of human rights conditions in mental health facilities by an independent body. 5/5 indicates that both the policies and laws are fully in line. Only Ireland admitted that their laws were not fully compliant, giving themselves a score of 5/3. The US did not score itself on laws, probably because each state has its own laws.

**Independent body monitors human rights?**  Countries could answer no or yes, with explanations of how often they were monitored.

**Formal collaboration with service users and family caregiver advocacy groups:** This question was asked in 2014 and 2017, but in 2020 it was folded into the question about policy and legislation being in line with human rights.

Indicators or targets against which policy plans can be monitored? This question was asked for the first time in 2020. The full question is “The mental health policy / plan contains specified indicators or targets against which its implementation can be monitored”

**Numbers of Psychiatrists, mental health nurses, psychologists, social workers and total mental health professionals per 100 K population**: The WHO Atlas defines the following:

**“Nurse:** A health professional who has completed formal training in nursing at a recognized, university-level school for a diploma or degree in nursing.

**Occupational therapist:** A health professional who has completed formal training in occupational therapy at a recognized, university-level school for a diploma or degree in occupational therapy.

**Other specialized mental health worker:** health or mental health worker who possesses some training in health care or mental health care (e.g. occupational therapist) but does not fit into any of the defined professional categories (e.g. medical doctors, nurses, psychologists, social workers). **Includes:** Non-doctor/non-nurse primary care workers, psychosocial counsellors, auxiliary staff. **Excludes:** General staff for support services within health or mental health care settings (e.g. cooking, cleaning, security).

**Primary health care doctor:** A general practitioner, family doctor or other non-specialized medical doctor working in a primary health care clinic.

**Primary health care nurse:** A nurse working in a primary health care clinic.

**Psychiatrist:** A medical doctor who has had at least two years of postgraduate training in psychiatry at a recognized teaching institution. This period may include training in any subspecialty of psychiatry.

**Psychologist:** A professional who has completed formal training in psychology at a recognized, university-level school for a diploma or degree in psychology. The Mental Health Atlas asks for information only on psychologists working in mental health care.

**Social worker:** A professional who has completed formal training in social work at a recognized, university-level school for a diploma or degree in social work. The Mental Health Atlas asks for information only on social workers working in mental health care.

**Speech therapist:** A professional who has completed formal training in speech therapy at a recognized, university-level school for a diploma or degree in speech therapy. In some countries, speech therapy is a part of audiology training. The Mental Health Atlas asks for information only on speech therapists working in mental health care.”

**Number of visits in hospital-based outpatient facilities, community based non-hospital outpatient facilities, Mental hospital beds, General hospital psych unit beds, Community residential beds, admissions to Mental hospitals.:** See definitions of facilities above.

**Involuntary admissions:** These have not been defined by the WHO, but we think that involuntary admissions should include any admissions that are not requested by the person hospitalised.

**Treated prevalence of psychosis:** the number of people per 100 000 population who have received care from inpatient or outpatient mental health facilities over the previous year for psychosis.

*OECD Benchmark for Mental Health Systems (25)*

The OECD Benchmark was prepared with the aim of helping countries to deliver high-performing mental health systems. It contains 23 indicators to assess the countries’ performance. It is also hampered by poor data availability. Only two indicators—Life satisfaction, and death by suicide—were available in more than 90% of countries. Like the WHO Mental Health Atlas, the data were obtained by Questionnaires, in this case to delegates of the OECD Health Committee. The benchmark indicators that we examined are reported in the supplemental spreadsheet tab “WHO vs OECD reports”. The columns, indicators and their explanations in the OECD Benchmark report are:

**Government expenditure on mental health care as a percent of health care expenditure:** From their Table 1.3: The footnote in the table state that some of this was taken from the OECD questionnaire, and some from the WHO Mental Health Atlas.

**Suicide Rates:** From Table 1.2 in OECD report, which was taken from OECD health statistics.

**Psychiatrists, Psychologists and Nurses:** From Table 1.3, again from the OECD questionnaire, and the WHO Mental Health Atlas, and National Sources.

Seclusion and restraint yearly numbers came from the OECD questionnaire. Types of restraints were not distinguished.

1. Australian Government. Mental health services in Australia-Restrictive Practices. In: Australian Institute of Health and Welfare, editor. [www.aihw.gov.au/mhsa](https://vuw-my.sharepoint.com/personal/savagema_staff_vuw_ac_nz/Documents/ZZ_MyHDocs/personal/kelly_savage/activism/oia_requests/our_restraints_paper/coercion_paper_2022/resubmission/new_revisions_july2023/www.aihw.gov.au/mhsa), 2016-2017.

2. Australian Institute of Health and Welfare. Mental Health Services in Brief, 2017. Canberra, Australia: Australian Institute of Health and Welfare; 2017.

3. Poupart T. Mental Health Bulletin: 2017-2018 Annual Report Reference tables. 2018. In: Health and Social Care Information Centre CMHT, NHS Digital [Internet]. England: England Government Statistical Service.

4. World Health Organisation. Mental health atlas 2020. In: World Health Organiszation, editor. Geneva, 2021.

5. Lepping P, Massood B, Flammer E, Noorthoorn EO. Comparison of restraint data from four countries. Social psychiatry and psychiatric epidemiology. 2016;51(1):1301-9.

6. Commission MH. The use of restrictive practices in approved centres: seclusion, mechanical and physical restraint activity report 2020. In: Commission MH, editor. Ireland: Mental Health Commission (Ireland); 2021.

7. Daly A, Walsh D. Activities of Irish Psychiatric Units and Hospitals, 2014. In: HRB Statistics, editor. Dublin: Health Research Board; 2015.

8. Anonymous. The "630" report survey results: FY2017-2022. In: Health Labor Sciences Research Grant, editor. Mental Health Welfare Materials: Japanese Ministry of Health Labor and Welfare; 2017-2023.

9. Newton-Howes G, MK S, Arnold R, Hasegawa T, Staggs V, S K. The use of mechanical restraint in Pacific Rim countries: an international epidemiological study. Epidemiology and Psychiatric Sciences 2020;29:1-7.

10. Yamanouchi Y. Report on the Research Project for Establishing an Effective Monitoring System for Medical Care Plans and Disability Welfare Plans (Mental Disability) (in Japanese). In: Research DoPP, editor. National Institute of Mental Health, National Center of Neurology and Psychiatry: National Institute of Mental Health; 2021.

11. Fukasawa M, Miyake M, Suzuki Y, Fukuda Y, Yamanouchi Y. Relationship between the use of seclusion and mechanical restraint and the nurse-bed ratio in psychiatric wards in Japan. International journal of law and psychiatry. 2018;60:57-63.

12. Hasegawa T. Survey of seclusion and restraint in psychiatric care: Investigating the background factors for the rapid increase and considering the path to reduction. (in Japanese). Japanese Journal of Hospital and Community Psychiatry (病院・地域精神医学). 2016;59(1):18-21.

13. Sugiura K, Morita Y, Kawakami N, Kayama M. How Do Psychiatrists in Japan Choose Involuntary Admission, and What Do They Think of Supported Decision Making? A Thematic Analysis of Peer to Peer Interviews. Community Mental Health Journal. 2022.

14. Anonymous. landelijke kerncijfers vrijheids-beperkende interventies 2013, in English: national key figures freedom-restricting interventions. Casusregister Argus; 2013.

15. Noorthoorn E, Voskes Y, Janssen W, Mulder C, van de Sande R, Nijman HL, et al. Seclusion Reduction in Dutch Mental Health Care: Did hospitals meet goals set? Psychiatric Services. 2016;1(12):1321-7.

16. Noorthoorn E, Lepping P, Janssen W, Hoogendoorn A, Nijman H, Widdershoven G, et al. One-year incidence and prevalence of seclusion: Dutch findings in an international perspective. Social Psychiatry and Psychiatric Epidemiology. 2015;50(12):1857-69.

17. Janssen W, van de Sande R, Noorthoorn E, Nijman H, Bowers L, Mulder C, et al. Methodological issues in monitoring the the use of coercive measures. Int J Law Psychiatry 2011;34(6):429–38.

18. New Zealand Ministry of Health. Mental Health and Addiction: Service use 2016/2017,. In: Mental Health and Addiction Programme for the Integration of Mental Health Data, editor. New Zealand: New Zealand Ministry of Health; 2021.

19. Medicare Services. Inpatient psychiatric facility quality reporting program manual. In: Centers for Medicare & Medicaid Services, editor. online, 2017.

20. Medicare. Veterans Health Administration Behavioural Health Data, 2018. 2019.

21. Staggs V. Predictors of seclusion and restraint following injurious assaults on psychiatric units. Journal of Patient Safety. 2021;17:562-7.

22. Lutterman T, Shaw R, Fisher W, Manderscheid R. Trend in psychiatric inpatient capacity, United States and each State, 1970-2014. In: Directors SMHP, editor. Alexandria, Virginia, USA: National Association of State Mental Health Program Directors; 2017.

23. Government of Wales. Admission of patients to mental health facilities: April 2020 to March 2021. In: gov.wales, editor. 2022.

24. Organization. WH. Global Health Observatory. 2019.

25. OECD. A New Benchmark for Mental Health Systems: Tackling the Social and Economic Costs of Mental Ill-Health,. Paris: OECD Publishing; 2021 8 June 2021.
